# Medical Society of the County of Erie

**Published:** 1905-07

**Authors:** Franklin C. Gram


### Medical Society of the County of Erie.

*Eighty-fourth Semiannual Meeting, June 13, 1905.*

Reported by FRANKLIN C. GRAM, M. D., Secretary.

THE president, Dr. John D. Macpherson, opened the eighty-fourth semi-annual meeting of the Medical Society of the County of Erie in the rooms of the Buffalo Society of Natural Sciences, at 10 a. m., June 13, 1905.

The minutes of the annual meeting and of the special meeting, held February 27, 1905, were read by the secretary and approved.

Dr. William Warren Potter, chairman of the membership committee, reported favorably the name of Dr. Myrtle Lothrop Massey, membership to date from January, 1905. The report was adopted.

Dr. Henry R. Hopkins, chairman of the board of censors, made a verbal report of the work performed by the censors during the past six months. Among other things accomplished the board has obtained the conviction of Louisa Busch for violation of the practice law. She was fined $250, and the fine was duly paid to the treasurer of the society. He offered a resolution that the secretary be directed to turn over to the censors all papers pertaining to the case of C. H. Woodward.

The censors' report was received and the resolution adopted.

Dr. Hopkins then submitted a report as chairman of the legislative committee and closed by offering the following:

Whereas, The Medical Society of the County of Erie holds the following truths to be axiomatic: that the preservation of the public health is the most important duty of the state: that an efficient medical profession is necessary to the existence of civilised life: that the medical profession must be organised in order to act with efficiency in matters of general or public interest,—

that the medical profession of this state should support the present standard of medical education required by our system of state examination,—that our system of state examination is almost invaluable though faulty in that we have three state examining boards where there should be but one. Therefore,

*Resolved,* That this society hereby instructs its committee on legislation to prepare a bill for the purpose of improving our system of state examinations—by creating a single examining board of such number of members, so selected as shall be determined by the various representatives of the medical profession.

*Resolved,* That the Medical Society of the County of Erie, State of New York, hereby authorises its committee on legislation with full power, and instructs the same to invite conferences with other societies, state and county, to the end that the proposed bill may meet the views, and have the support of the medical profession of the state of New York.

Dr. William Warren Potter took exception to the wording of the resolution. He said it was in this body that the present state medical practice law had its inception and was referred to the medical society of the state where it was deliberated, and fought over for seven years in the legislature before receiving favorable action. When it became apparent that no single board could be had the committee said to the legislature, "Give us as many examining boards as you like, but make the standard uniform and we will be satisfied," whereupon three boards were created. There may be good arguments, he said, for or against three examining boards, and he moved as an amendment that the proposition be referred to a select committee of five to confer with other medical organizations, which are parties in interest, before giving instructions for the preparation of a bill.

Dr. Wende: "Is not that embodied in Dr. Hopkins's resolution?"

Dr. Hopkins read his resolution again and spoke against Dr. Potter's amendment, because the legislative committee of which he is chairman, could deal with it. He said that perhaps his phraseology had been unfortunate and he asked permission to amend.

Dr. Potter: "If I understand the resolution, it instructs the committee to prepare a bill. It seems to me the conferences should be had before instructions are given to prepare a bill."

Dr. Hopkins: "The intention is that conferences shall be had before a bill is prepared."

Dr. Potter accepted this explanation and withdrew his amendment.

The resolution was then adopted.

Dr. William Warren Potter called attention to the Association of Military Surgeons of the United States, incorporated by act of congress. Its president is Surgeon General Walter Wyman, and the first vice-president is our distinguished citizen and vice-president of our county society, Lieut. Col. A. H. Briggs. The next meeting will be held in Detroit, September, 1905, and in the ordinary nature of affairs it may be expected that Dr. Briggs will be elevated to the presidency. He, therefore, moved that Dr. Briggs be authorised to invite, on behalf of this society, the Association of United States Military Surgeons, to hold the following annual meeting (1906) in Buffalo.

The motion was carried unanimously.

Dr. Frederick H. Millener read a paper on

PERNICIOUS EFFECTS OF ALTERNATING CURRENT OF HIGH VOLTAGE.

(See page 788.)

This paper was discussed by Dr. Briggs who expressed the opinion that practitioners should inform themselves on this subject, because they are called upon to deal with the results of electricity.

Dr. J. Henry Dowd read a paper on

IRRITABLE BLADDER.

Dr. Hopkins, in discussing the paper, said that the drinking of too much water caused more irritable bladders than all the gin, whiskey, and beer on the market. Every person could drink before breakfast all the water required by the system for the entire day.

Dr. Eli H. Long asked Dr. Hopkins to give a standard for the amount to be drank.

Dr. Hopkins: "Under ordinary circumstances one pint daily is sufficient, extraordinarily two pints."

Dr. Long: "Would you drink that amount before breakfast?"

Dr. Hopkins: "If you chose."

Dr. Benedict said he would rather have an irritable bladder through drinking water, than have barnacles on his bowels by not drinking enough.

Dr. Dowd insisted that the amount of urine voided had no relation to the amount of water drank by the individual, and cited instances in which the patients drank no water, yet passed excessive quantities of urine.

Dr. H. R. Gaylord spoke on "The Present Status of Kidney Surgery."

President Macpherson thanked the various contributors for their interesting efforts.

Dr. William C. Krauss said that many had enjoyed last year's excursion, and stated that inquiries had been made for holding another at a proper time this summer.

On motion the president appointed Drs. Krauss, Hopkins, and Briggs a committee with power in this matter.

The society then adjourned.

---

Inflammable Flannelette.—Surely the days of inflammable flannelette should be speedily numbered. The death roll among children who have been fatally injured by the ignition of this perilous fabric is simply appalling. The wearing of flannelette has again and again exposed children to the same risk as if their night dresses were soaked in spirit. The fabric catches fire as easily and burns with the same intense flame as alcohol, and the flames are not readily extinguished. "An inquest was held recently on the body of a little boy 2 year old. He was left to play in a room while his mother was absent. He was in a flannelette nightshirt. The mother had not left the room long when she heard screams and found the boy in flames. He was terribly burned, and the poor little fellow died within twenty-four hours of the occurrence." Such is the sort of heartrending paragraph constantly appearing in the newspapers. One coroner alone has stated that last year he held no less than seventy-three inquests on children who had been burned to death, and a large proportion was due to flannelette igniting.—*The Lancet.*

---

Professor Brieger, of the Berlin Medical Institute, was busily at work in his laboratory surrounded by a formidable array of chemical and bacteriological utensils. A distinguished foreign physician called upon him and watched his absorbing labor with interest.

The professor's attention seemed to be anxiously, but still hopelessly, concentrated on a vessel which was enveloped in smoke and steam.

"Guess what I am boiling here in this pot," said the professor.

The visitor began to enumerate the entire scale of microörganisms.

"Micrococci?"

"No."

"Sonococci?"

"No."

"Spirochaeta?"

"No."

"What then?"

"Sausages," replied Brieger.—*Cleveland Plain Dealer.*

